# Characterization of Soil Microbial Communities Associated with the Sustainability of Acacia Species in the Imam Turki Bin Abdullah Royal Reserve, Saudi Arabia

**DOI:** 10.3390/plants15193023

**Published:** 2026-10-02

**Authors:** Mohammed A. Alduwayghiri, Abdulaziz Alharbi, Medhat Rehan

**Affiliations:** 1 Department of Plant Production, College of Agriculture and Food, Qassim University, Buraydah 51452, Saudi Arabia; 451113842@qu.edu.sa; 2 National Center for Vegetation Cover Development and Combating Desertification, Riyadh 13312, Saudi Arabia; 3 Department of Environment and Natural Resources, College of Agriculture and Food, Qassim University, Buraydah 51452, Saudi Arabia; abb.alharbi@qu.edu.sa

**Keywords:** nature reserves, amplicon-sequencing, macro- and micro-elements, soil fertility, sustainability

## Abstract

The Imam Turki bin Abdullah Royal Reserve, one of Saudi Arabia’s largest protected areas, harbors extensive Acacia populations whose rhizosphere microbiota enhance nutrient availability and ecosystem sustainability. The present study aimed to characterize the soil and plant nutrient status alongside the composition of microbial communities of two Acacia species to support biodiversity conservation and desertification mitigation. Fifteen paired leaf and rhizosphere soil samples were collected from *Acacia gerrardii* Benth (*n* = 9) and *Acacia raddiana* Savi (n = 6). The two plant groups exhibited distinct macro- and micro-elemental profiles, indicating variations in nutrient availability across the sampled habitats. Amplicon-sequencing revealed diverse bacterial communities, with Actinobacteriota, Chloroflexi, and Proteobacteria representing the dominant bacterial phyla. The genus *JG30-KF-CM45* (Chloroflexi) was particularly abundant in several soil samples. Likewise, fungal communities were overwhelmingly dominated by Ascomycota, whereas Basidiomycota occurred at lower abundance. *Didymella*, *Pithoascus*, and *Fusarium* were among the most prevalent fungal taxa. Functional predictions indicated significant variation in enzymes associated with nutrient transformation and microbial metabolism, involving Zinc D-Ala-D-Ala carboxypeptidase, serine-type D-Ala-D-Ala carboxypeptidase, amidase, UDP-N-acetylglucosamine 1-carboxyvinyltransferase, and α-amylase. In addition, the KEGG Orthologs (KOs), K08640 (zinc D-Ala-D-Ala carboxypeptidase), K00657 (diamine N-acetyltransferase) and K06147 (ATP-binding cassette, subfamily B), had the highest relative abundances in bacterial communities. These findings highlight distinct rhizosphere microbial assemblages associated with Acacia species, which may contribute to plant performance and ecosystem functioning in arid environments.

## 1. Introduction

A desert ecosystem type nature reserve is one that prioritizes preserving the biotic and abiotic natural ecosystems of the desert. These reserves are located in delicate ecosystems that experience problems like drought, overgrazing, and land degradation [1,2]. Enhancing the administration and safeguarding of these reserves is therefore essential. The United Nations’ 2030 Agenda for sustainable development calls for members to protect, restore, and promote sustainable land use through ecological compensation in order to combat desertification by limiting and reversing land degradation [3].

In Saudi Arabia, the protected areas are diverse and strategically established to protect the country’s ecosystems. The reserves vary in terms of size and ecological focus, with an emphasis on maintaining both plant and animal biodiversity. There are notable protected areas such as Prince Mohammed bin Salman Royal Reserve that focuses on the protection of desert plants such as Ghada (Tamarix) and Arta (Salsola), whereas King Abdulaziz Royal Reserve aims to rehabilitate degraded desert landscapes and increase the population of native plants while enhancing biodiversity [4]. Imam Turki bin Abdullah Royal Reserve considers one of the largest and most significant reserves in Saudi Arabia that focuses on rehabilitating ecosystems by planting native species, enhancing biodiversity, and maintaining ecological balance. It locates in the northeastern part of Saudi Arabia and dedicated to conserve the region’s biodiversity. Spanning over 91,000 square kilometers, the reserve consists of various landscapes, including sand dunes, valleys, and plateaus [5,6].

Role of plants in protected areas involves: (1) soil improvement via enhance soil fertility and prevent erosion caused by wind and water through root systems that help stabilize soil and prevent degradation; (2) supporting wildlife through providing food and shelter for numerous animal species and creating a balanced ecosystem; (3) combating desertification in the arid regions since native plants stabilize sandy soils and reduce the impacts of wind erosion; (4) ecological sustainability through maintaining plant diversity, thus ensuring the health and functionality of the ecosystem [4,7,8,9].

The majority of Acacia species are found in arid and semi-arid climates worldwide. In the world’s warm and tropical climates, Acacia species are significant both socially and economically. These species are said to be the most effective trees in desert areas because they can tolerate severe weather [10]. In some regions of Saudi Arabia, Acacia species are naturally found as woodlots. In Saudi Arabia, a nation with vast stretches of desert and very little vegetation cover, Acacia spp. are particularly important species of flora. Acacia communities have a high cover and minimal species variety and consider the pinnacle of xerophytic vegetation in Saudi Arabia [11,12]. The majority of the floral composition in the southwestern highlands comprises Acacia species. Al-Ghamdi [13] stated that this area accounts for around 70% of the nation’s beekeeping output, where these plant species are essential to the flora of honey bees. *Acacia* spp. L. (Fabaceae) considers one of the dominant trees in the highlands of southwest Saudi Arabia [14,15]. According to Dharani [16], *Acacia gerrardii* is indigenous to subtropical areas of western Asia and Africa and consider one of the most prevalent Acacias on the Arabian Peninsula. *Acacia gerrardii* is a tiny, evergreen tree that grows to a height of 5 to 10 m. Its rounded crown, which is typically greater in diameter than it is in height, offers shade to livestock and wildlife. The plant is well-suited to withstand drought stress because it has a deep taproot, lives in open woodlands, arid and semi-arid savannahs on well-drained soil, and can withstand a wide range of environmental conditions [17,18,19].

Soil microorganisms in the rhizosphere are essential for preserving soil biochemistry and ecosystem function through the breakdown of organic matter, the cycling of nutrients, and the production of humus. Tropical soil ecosystems are distinguished from temperate counterparts by their rich tapestry of diversity and complexity [20]. Exploring desert soils, which are characterized by severe solar radiation, little precipitation, and temperature fluctuations, reveals the presence of highly varied bacterial communities. A robust and dry-adapted bacterial biodiversity coexists peacefully with plants in semi-arid tropical biomes where grassy vegetation predominates [20,21].

Microbial community diversity is frequently used to assess soil fertility and quality [22,23,24]. There are many different types of relationships between plants and soil-dwelling organisms, including exploitative, commensal, competitive, mutualistic, and neutral ones. Information about the diversity and structure of microorganisms is very important for managing land use and soil quality. Theoretical guidance for assessing and improving the soil environment can be obtained from the microbial populations in various land types [24,25,26]. Microbial activity can promote plant development through three mechanisms: (1) making soil-borne nutrients more bioavailable; (2) secreting plant hormones and controlling their signals; and (3) battling with harmful microbial strains [23,27,28,29]. Soil microbes solubilize inaccessible nutrients, making them more accessible to plants. Many of the nutrients in natural ecosystems, such as N, P, K and S, have low bioavailability for plants because they are bonded in organic substances. Soil microbes such as bacteria and fungi have the metabolic machinery to solubilize and/or mineralize the organic forms of N, P, K and S required for plant growth [30]. Inorganic N, P, and S forms as well as ionic species including nitrate, ammonium, sulfate, and phosphate will be released into the soil and absorbed by plants depending on the microorganisms’ activities [20,30,31,32,33,34].

Amplicon-sequencing is recognized as a valuable technique for studying soil health by identifying microbe communities and their functional possibility. This strategy includes sequencing of highly conserved regions in microbes present within a soil sample, giving insights into the biodiversity, composition, and functional properties of the soil microbiome. The technique of sequencing analysis based on the conserved regions in the 16S rRNA (V3–V4 regions) and ITS has been widely employed in recent years to detect the microbiome due to the quick development of next-generation sequencing and molecular biology [35,36,37]. The major objectives of the present research were as follows: (1) assess the nutritional status of Acacia plants and soil properties in the root zone in Imam Turki bin Abdullah Royal Reserve; (2) identify the microbial communities associated with the root zone of Acacia plants; (3) characterize the soil and plant nutrient status as well as the composition of microbial communities across the studied ecosystems and assess their potential contributions to ecosystem sustainability and desertification mitigation.

## 2. Results

### 2.1. Soil and Plant Contents from Nutrient Elements

The available soil nutrients in the rhizosphere area of *Acacia gerrardii* Benth (group 1) and *Acacia raddiana* Savi (group 2) plants in the Imam Turki bin Abdullah (ITBA) Royal Natural Reserve, were evaluated and summarized in Table 1. The nutrient elements exhibited significant differences among the assessed soil samples. Regarding the macro-elements, the nitrogen (N) element presented non-significant differences between the two soil groups with uppermost percentages 0.15 ± 0.02% in group 1 soil, whereas soil of group 2 displayed lower nitrogen content (0.13 ± 0.03%). In addition, Phosphate (P) revealed significant and higher content in soil of group 2 (21.93 ± 8.17 ppm) when compared with group 1 soil (11.1 ± 7.82 ppm, *p* < 0.05). With average content measured 71.83 ± 34.28 ppm in group 2 soil and 51.67 ± 15.51 ppm in group 1 soil, potassium (K) content had no significant differences among the two soil groups. Furthermore, the higher and significant availability of micro-nutrients such as Mn, Zn and Cu was observed in soil of group 2 plants with values reached 56.41 ± 9.81, 25.29 ± 4.86 and 6.11 ± 0.71 ppm, respectively. Meanwhile, the maximum value from Fe (3304.78 ± 382.35 ppm) was found in group 2 soil without significant differences when compared with group 1 soil.

The available plant nutrients in *Acacia gerrardii* Benth (group 1) and *Acacia raddiana* Savi (group 2) were also analyzed and assessed for possible relationship among the soil, plant elements content and the soil microbiota (Table 2). The macro-elements (N, P and K) were detected in higher amount in group 1 plants with values calculated 2.47 ± 0.65%, 1010 ± 390 ppm, and 6920 ± 100 ppm, respectively, without significant differences among groups. When going forward and evaluate the micro-elements in plant samples, the best value from iron (Fe) was found in group 1 plants (170.55 ± 44.88 ppm), whereas zinc (Zn), and copper (Cu) presented the non-significant and the topmost available content (15.73 ± 3.5, and 7.4 ± 1.73 ppm) in plants of group 1, respectively. In addition, magnesium (Mg) displayed the maximum amount in group 2 plants (1990 ± 480 ppm).

Regarding Mn and Ca, group 1 plants (A1–A9) exhibited significantly higher Mn concentrations (32.3 ± 9.65 ppm) than group 2 plants (22.32 ± 5.03 ppm), while group 2 showed significantly lower Ca levels (5340 ± 170 ppm) compared with group 1 (6020 ± 450 ppm). Overall, the results suggest that the primary nutritional distinction between the groups lies in Mn enrichment and Ca accumulation, highlighting potential differences in nutrient cycling processes and plant nutrient-use efficiency.

### 2.2. Amplicon-Sequencing Data Processing and Statistics

The generated amplicons from 15 samples (nine samples assigned as group 1 and six samples named group 2) produces approximately 250 bp paired-end raw reads (Supplementary Materials: Tables S1 and S2). The produced clean tags varied between bacterial and fungal communities with minimal numbers (114,002 nt) as detected in sample A2 in fungal communities, whereas the topmost clean tags (443,996 nt) were found in sample A5 in bacterial communities. In addition, the percentages of quality score ≥ 30 (error rate < 0.1%) in bacterial communities ranged from 92.47% to 93.31%, whereas 94.13% to 95.35% were displayed in fungal communities.

### 2.3. Microbial Taxonomy Distribution

The composition and distribution of bacterial comminutes in the rhizospheric soil samples revealed 33 phyla, 96 classes, 224 orders, 362 families, 637 genera and 1194 species. Furthermore, the fungal communities’ composition displayed 9 phyla, 27 classes, 61 orders, 133 families, 264 genera and 381 species. Species-level assignments should be considered presumptive, because the taxonomic resolution of short 16S rRNA gene/ITS amplicons is necessarily constrained by marker length, reference database coverage, and classification criteria.

**Figure 1:**
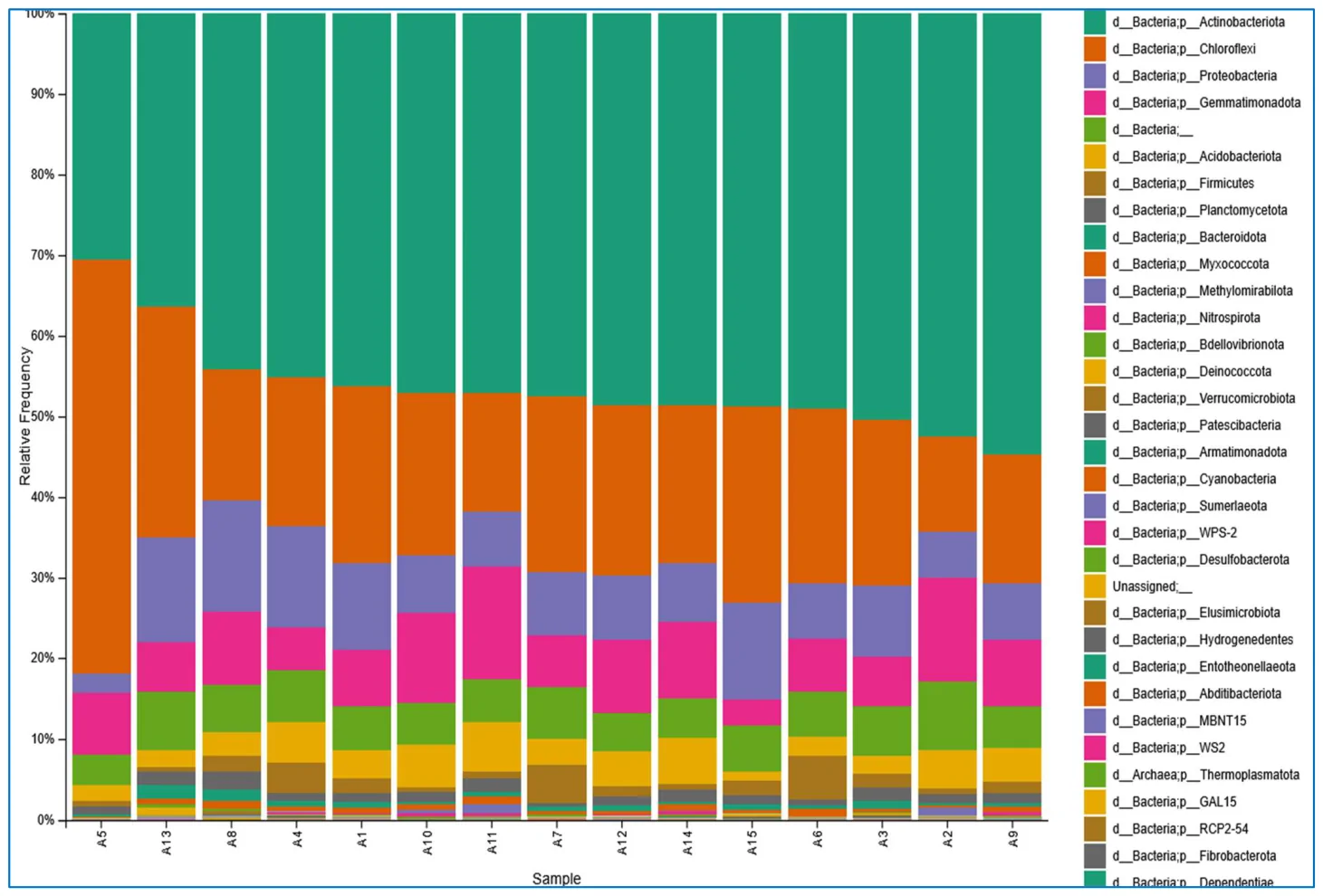
The bacterial distribution at phylum-level taxonomic in the rhizosphere soil samples of Acacia plants.

In the bacterial communities, the uppermost taxonomic distribution was assigned to Actinobacteriota at the phylum level, followed by Chloroflexi, Proteobacteria and Gemmatimonadota. The percentages of Actinobacteriota counted 54.68% in sample A9 and 52.55% in sample A2 from their bacterial population, whereas Chloroflexi displayed 51.31% of A5 and 28.61% from A13 populations (Figure 1). Proteobacteria considered the third abundant bacteria in the soil samples and presented 13.93% from the A8 population whereas Gemmatimonadota recorded the highest percentage in A11 sample and calculated 14.10% from its population. The genus *JG30-KF-CM45* (belonging to phylum Chloroflexi) was the predominant bacterial taxon across all soil samples, except for sample A2, where the most abundant taxon was an unclassified bacterium (8.5% relative abundance). The relative abundance of *JG30-KF-CM45* ranged from 8.19% in sample A8 to 36.0% in sample A5, highlighting its widespread distribution and ecological dominance across the studied soil microbiomes (Supplementary File S1).

**Figure 2:**
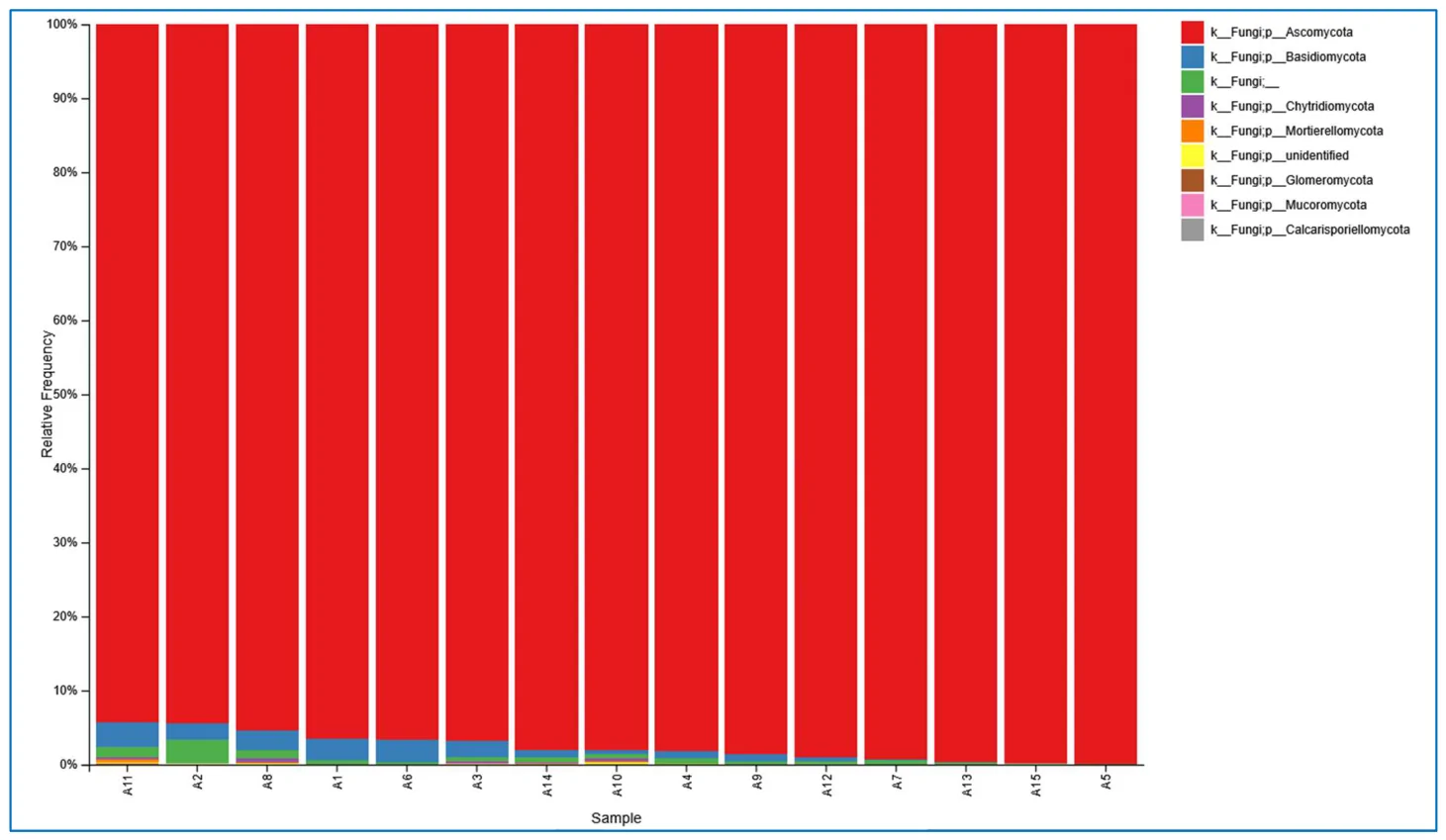
The phylum-level taxonomic distribution of fungi in rhizosphere soil samples of Acacia plants.

Likewise, the fungal microbial communities abundance varied in the assessed samples and Ascomycota in addition to Basidiomycota recorded the maximum enrich phyla. Ascomycota had the dominant phylum in all evaluated samples with more than 99% in samples A5, A15, A13, A7 and A12 (Figure 2). In the same manner, Basidiomycota showed the second common phylum abundance with 3.28% and 2.99% in samples A11 and A6, respectively. When evaluate the fungal communities richness, family Microascaceae represented the maximum percentage in A10 sample (16.5%), whereas the genus *Didymella* and *Pithoascus* calculated the topmost fungal content in A12 and A15 (17.3%, 54.6%), respectively (Supplementary File S2). The *Fusarium* and *Didymella* counted the highest fungal genera in A2 and A12 samples (18.75% and 17.3%), respectively.

### 2.4. Microbial Abundance Heatmap

**Figure 3:**
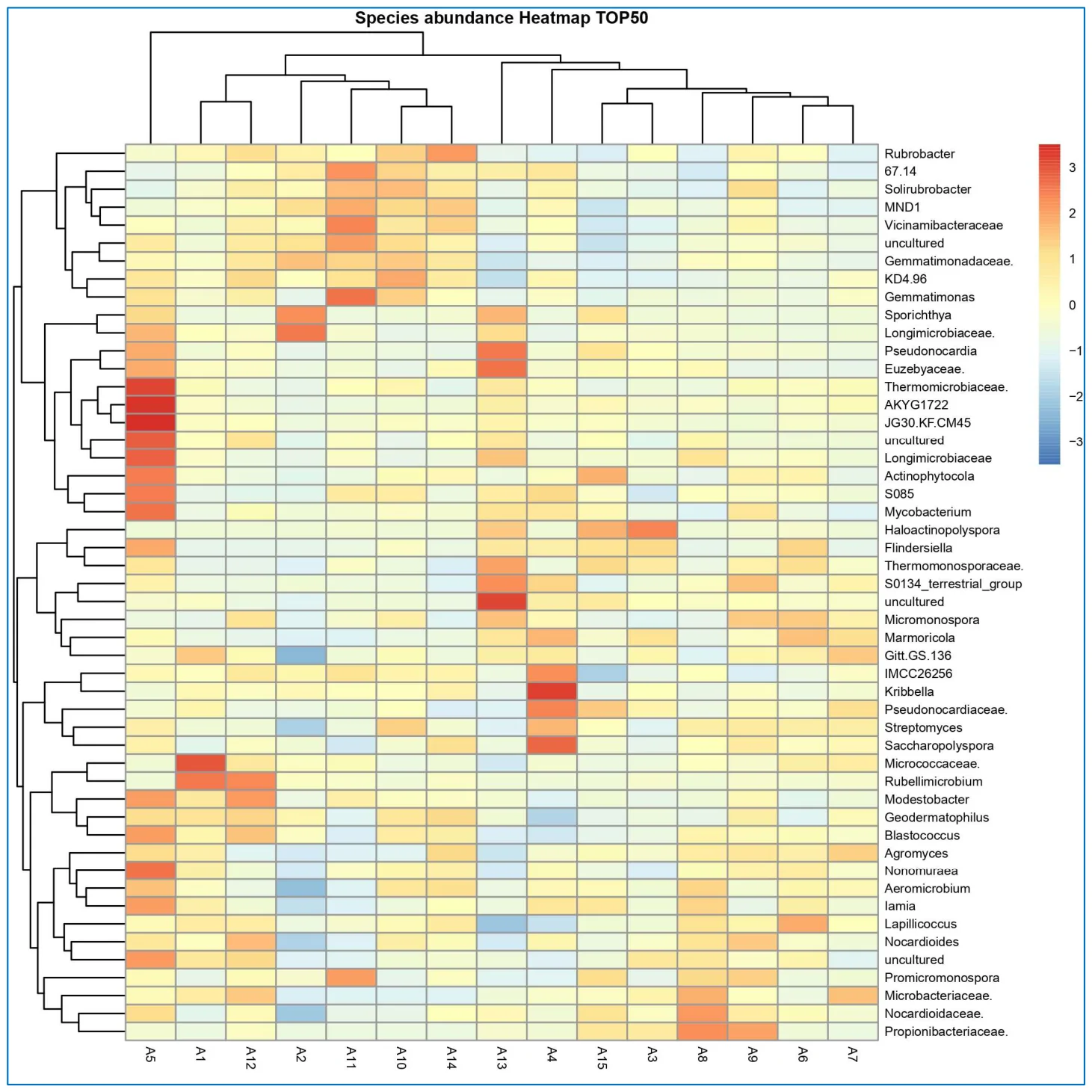
The bacterial composition heatmap at genus level, generated from the rhizosphere soil samples of Acacia plants.

The taxonomic content and relative abundance of each sample were used to perform heatmap analyses at the taxonomic genus level. After choosing the top 50 genera, samples and relative abundant species were grouped to create the heatmap. Figure 3 represents that samples were grouped in two main groups, the 1st group contained A7, A6, A9, A8, A3, A15, A4 and A13, while the 2nd group involved A14, A10, A11, A2, A12 and A1. Otherwise, A5 was considered as out of group sample. The genera and families such as *Rubrobacter*, *Kribbella*, *Saccharopolyspora*, *Thermomicrobiaceae*, *AKYG1722*, *Gemmatimonas*, *JG30-KF-CM45*, uncultured, *Micrococcaceae*, *Longimicrobiaceae*, *Actinophytocola*, *S085* and *Mycobacterium* were the most relative enrichment patterns across the assessed samples (i.e., A1, A4, A5, A11, and A13). Differential abundance analysis identified nine taxa with nominally significant differences between the two sampled Acacia populations (raw *p* < 0.05), although none remained significant after Benjamini–Hochberg correction (FDR > 0.05).

**Figure 4:**
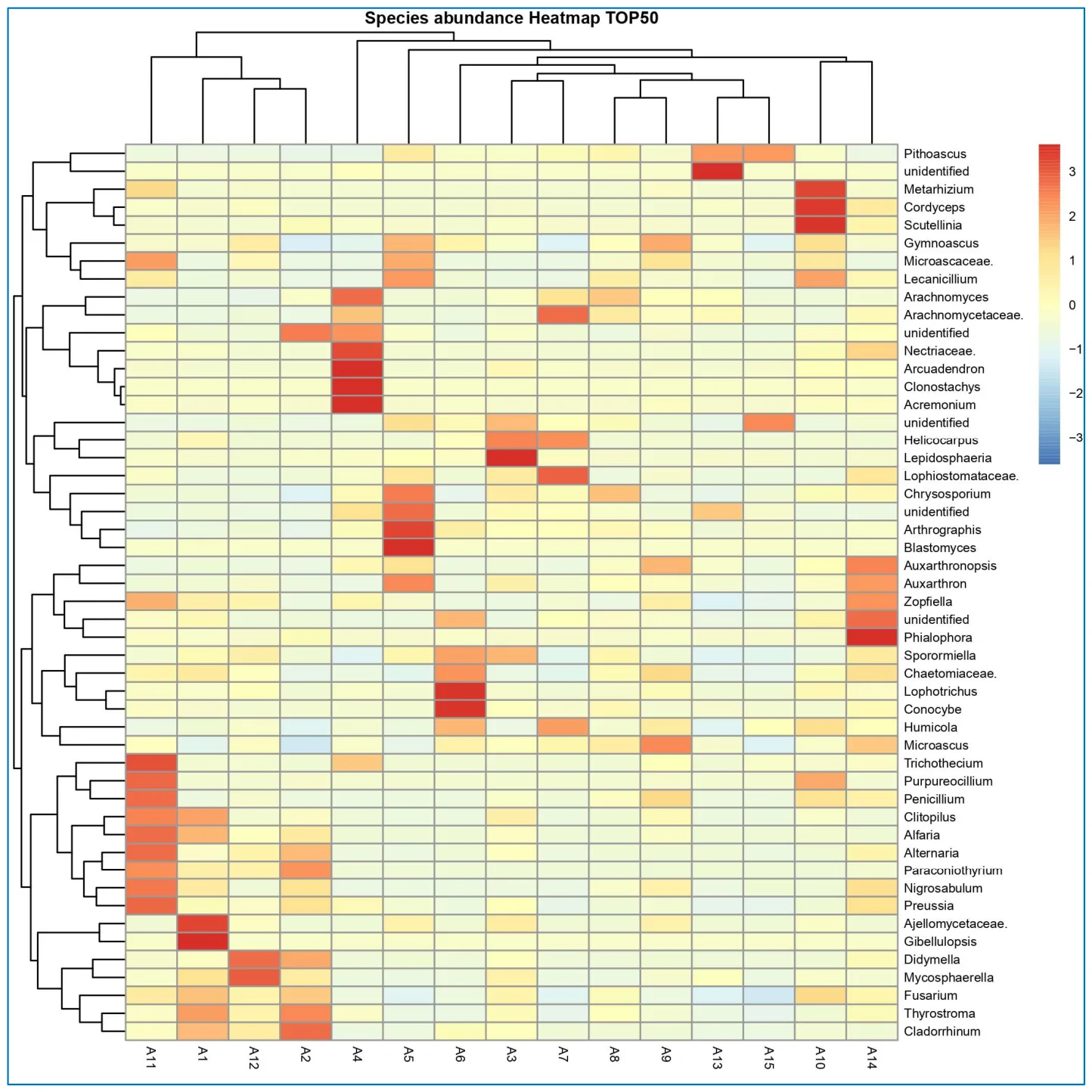
The fungal composition heatmap at genus level, generated from the rhizosphere soil samples of Acacia plants.

When going forward and explore the fungal community composition, the soil samples were categorized into two main groups based on their fungal content (Figure 4). One group involved samples such as A14, A10, A15, A13, A9, A8, A7, A3, A6, A5 and A4, whereas samples A1, A2, A11 and A12 were assigned to the second group. Furthermore, the fungal genera and families like *Metarhizium*, *Cordyceps*, *Scutellinia*, unidentified, *Nectriaceae*, *Arcuadendron*, *Clonostachys*, *Acremonium*, *Lepidosphaeria*, *Arthrographis*, *Blastomyces*, *Phialophora*, *Lophotrichus*, *Conocybe*, *Trichothecium*, *Ajellomycetaceae* and *Gibellulopsis* displayed the maximum relative enrichment microflora in all soil samples (i.e., A10, A14, A13, A3, A6, A5, A4, A11 and A1). There were no significant differences in fungal genera between group 1 (A1–A9) and group 2 (A10–A15) after FDR correction (q > 0.05 for all taxa). The genus *Cordyceps* had the strongest unadjusted group difference (H = 9.46, *p* = 0.0021, FDR = 0.556), but this result was not significant after multiple-testing correction (q > 0.05).

### 2.5. Diversity of the Microbial Communities

#### 2.5.1. Alpha Diversity

**Figure 5:**
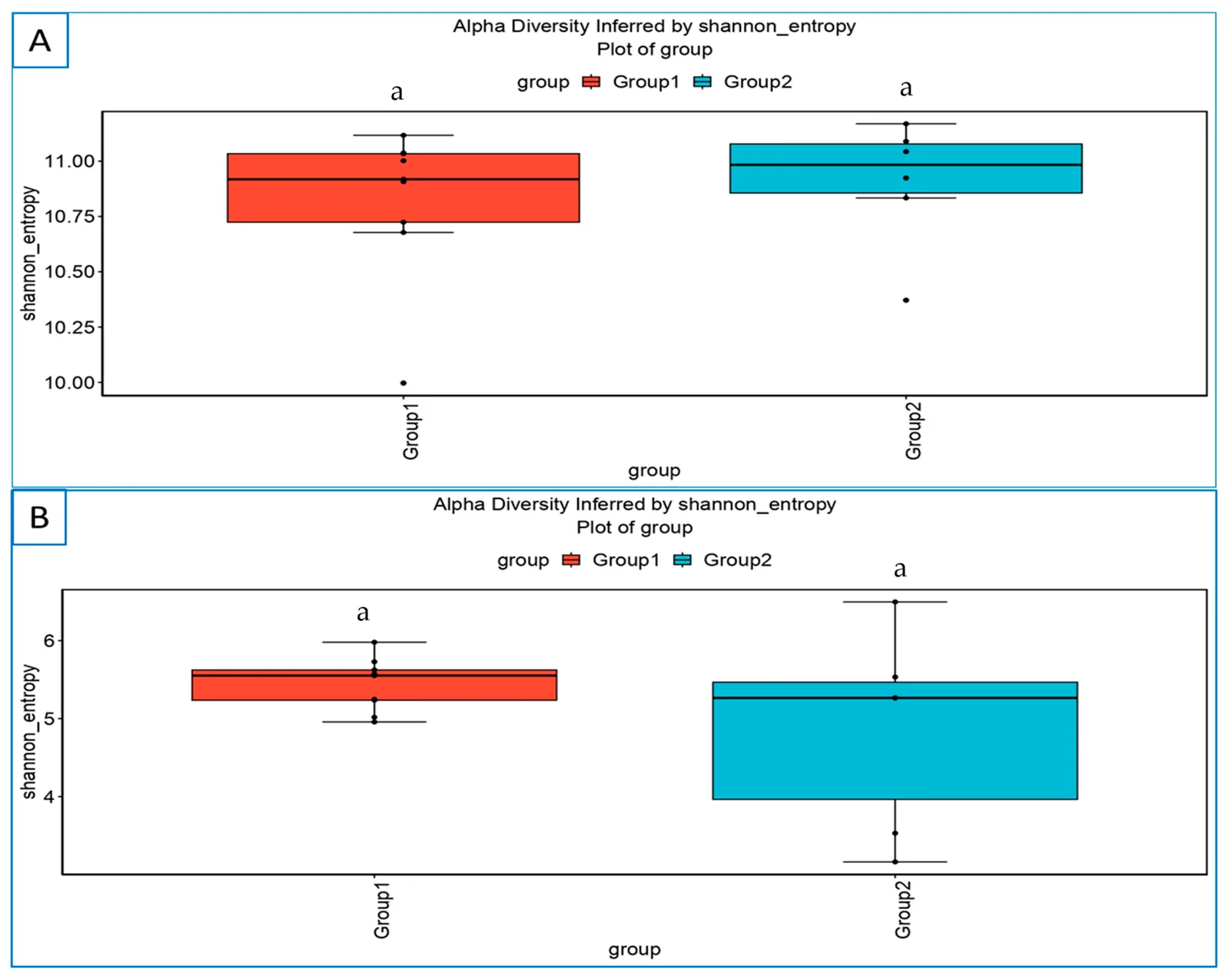
Alpha diversity inferred by Shannon entropy plot between (**A**) two groups of bacterial communities, and (**B**) two groups of fungal communities of Acacia plant soil (red = group 1 and blue = group 2). The same letters mean no significant differences (*p* ≤ 0.05).

To assess the microbial diversity, α-diversity indices was inferred using Shannon entropy index (Figure 5). In bacterial communities, Shannon diversity indices ranged from 9.99 (sample A5, group 1) to 11.17 (sample A12, group 2), indicating greater diversity and evenness in the bacterial communities. Although group 2 exhibited a slightly higher mean Shannon diversity than group 1 (10.91 vs. 10.82), no significant difference was detected between groups (Figure 5A). Furthermore, Shannon diversity indices ranged from 3.17 (sample A15) to 6.50 (sample A14) across fungal communities, displaying moderate to high fungal diversity among samples (Figure 5B). Group 1 showed a higher mean Shannon diversity than group 2 (5.44 vs. 4.88), suggesting a tendency toward greater fungal richness and evenness without significant differences. The lower Shannon values observed in some samples in group 2 represent a greater dominance of a limited number of fungal taxa, whereas higher values reflect more balanced community structures.

#### 2.5.2. Beta Diversity

**Figure 6:**
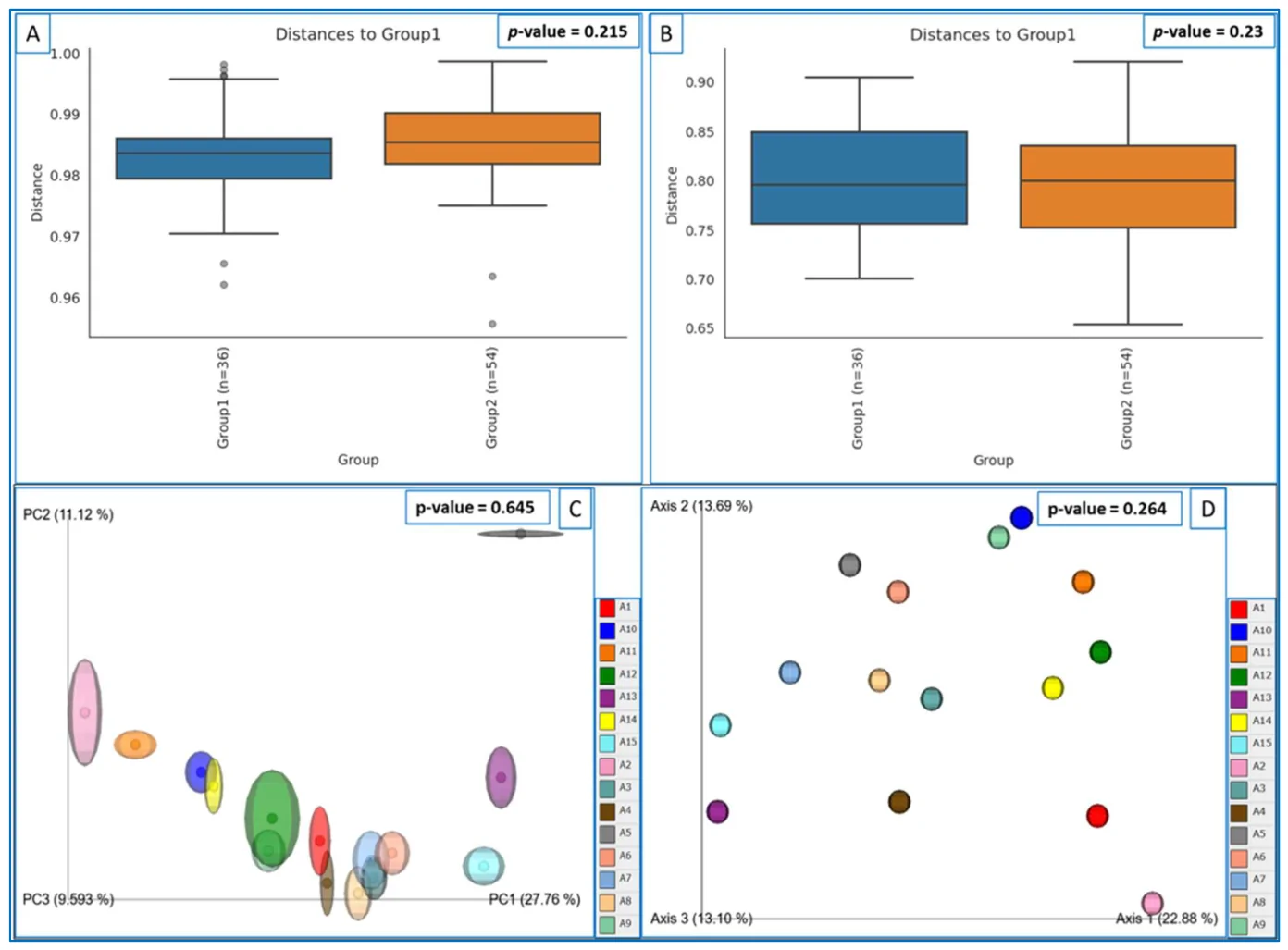
Beta diversity inferred by boxplot (jaccard) among bacterial and fungal communities (**A**) and (**B**), (red = group 1 and blue = group 2), and PCoA using weighted unifrac for bacteria communities (**C**) and Bray–Curtis for fungal communities (**D**), respectively, in Acacia plant soil.

Likewise, the beta diversity analysis was visualized using boxplot (jaccard) matrix to reflect the microbial composition among the two naturally occurring Acacia populations (Figure 6A,B). The associated bacterial species differed between the two Acacia groups with higher variation in group 2 in comparison with group 1. Conversely, fungal communities appeared relatively more variable in group 1 (*A. gerrardii*) than in group 2 (*A. raddiana*), suggesting broader compositional dispersion between the sampled populations. However, PERMANOVA analysis indicated that the overall multivariate community composition did not differ significantly between the groups for either bacteria (*p* = 0.215) or fungi (*p* = 0.230), suggesting that the observed variations were not sufficient to support statistically significant differences in community structure. The beta diversity was visualized also using PCoA (Principal Coordinates Analysis), based on UPGMA (weighted unifrac) to reflect bacterial community composition across soil samples (Figure 6C). PCoA revealed variation in bacterial community composition among samples, with the first three axes explaining 48.47% (PC1 = 27.76%, PC2 = 11.12%, and PC3 = 9.59%), of the total variance. Sample A5 exhibited a visible separation along the positive PC2 axis, whereas A13 and A15 were positioned toward the positive side of PC1, suggesting distinct community structures relative to the remaining groups. Bray–Curtis dissimilarity revealed variation in fungal community composition among samples (Figure 6D). The first three PCoA axes explained 49.67% of the total variation (PC1 = 22.88%, PC2 = 13.69%, and PC3 = 13.10%). Samples were dispersed across the ordination space, indicating differences in fungal assemblage structure and taxon abundance profiles. Samples A9 and A10 showed relatively close proximity, whereas A2 and A13 occupied more distinct positions, suggesting greater compositional divergence from the remaining samples.

Overall, PERMANOVA analysis displayed that microbial community compositions represented by this distance matrix do not differ significantly between group 1 and group 2 (PERMANOVA, pseudo-F = 0.85, *p*-value = 0.645 for bacterial community and pseudo-F = 1.15, *p*-value = 0.264 for fungal community, Figure 6C,D). To assess the homogeneity of multivariate dispersions between groups, PERMDISP was implemented using the same distance matrix and showed no significant differences in multivariate dispersion for either bacterial (F = 0.014, *p* = 0.911) or fungal communities (F = 0.211, *p* = 0.652), indicating similar within-group variability across treatments.

### 2.6. Functional Enzymes and Pathways Prediction

**Figure 7:**
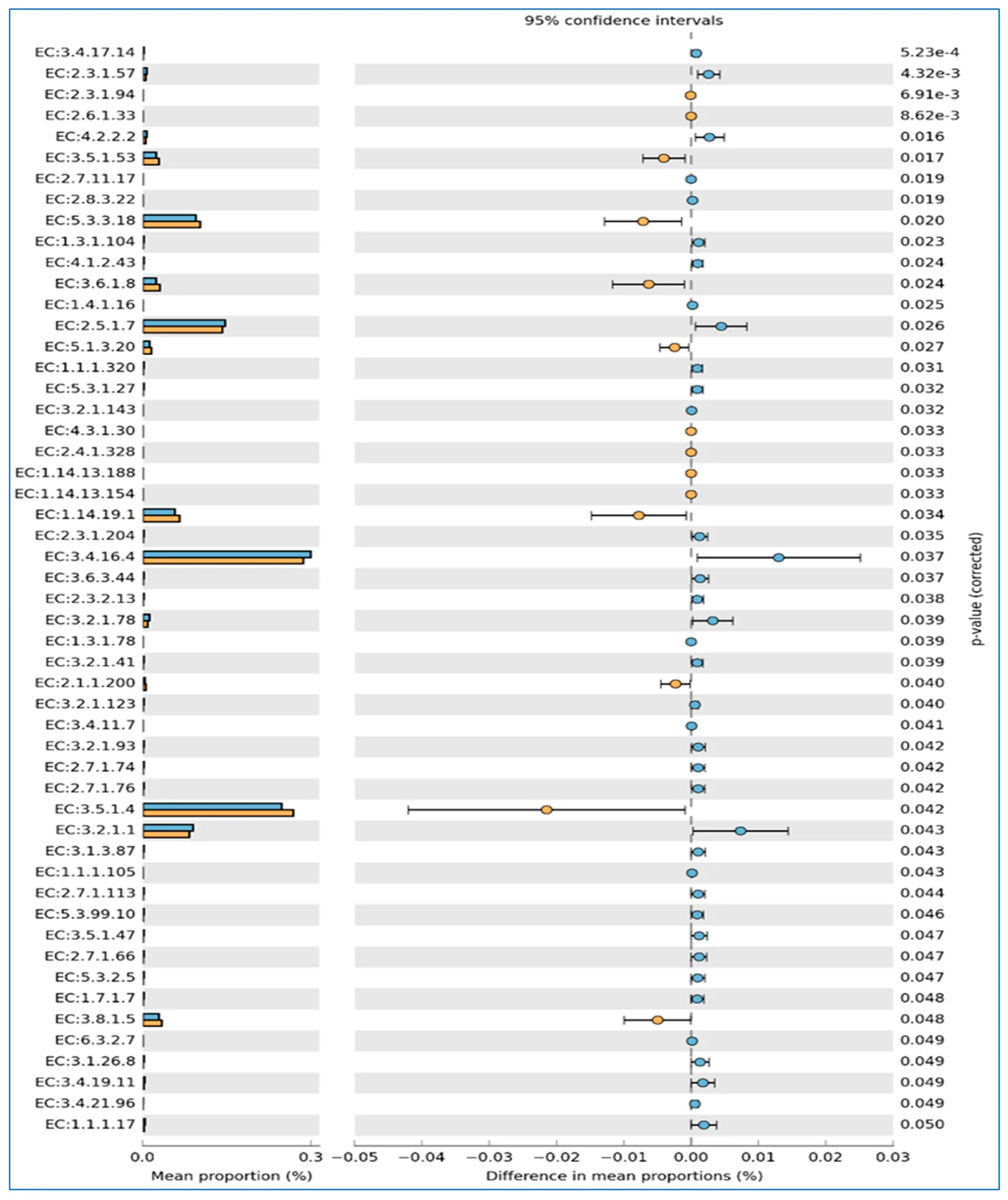
Differential abundance analysis of PicRust-predicted enzyme commission (EC) functions between bacterial groups (blue = group 1 and brown = group 2). The left panel shows the mean relative abundance (%) of each significantly or non-significant different EC category, while the central panel presents the difference in mean proportions with 95% confidence intervals. The right panel displays corrected *p*-values for each enzyme category.

The functional prediction based on PicRust, following multiple-testing correction, identified several enzyme commission (EC) categories that differed significantly between the compared bacterial communities (*p* ≤ 0.05). Among the significantly different enzymes in the bacterial communities, EC:3.4.17.14 (Zinc D-Ala-D-Ala carboxypeptidase), EC:2.3.1.57 (Diamine N-acetyltransferas), EC:3.4.16.4 (Serine-type D-Ala-D-Ala carboxypeptidase), EC:3.5.1.4 (Amidase), EC:2.5.1.7 (UDP-N-acetylglucosamine 1-carboxyvinyltransferase), and EC:3.2.1.1 (Alpha-amylase) showed the most statistically significant and the highest relative predicted abundances with the largest potential function alterations (Figure 7).

**Figure 8:**
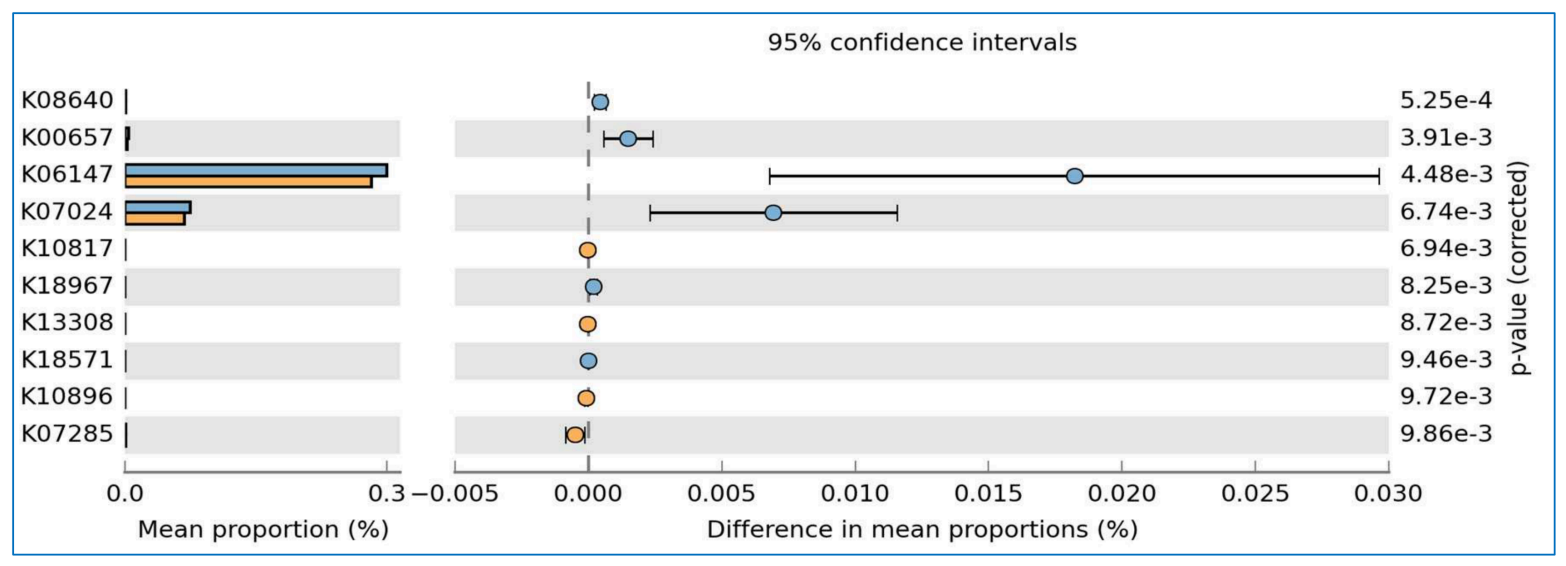
Differential abundance of significantly enriched KEGG orthologs (KOs) between the studied two bacterial communities groups (blue = group 1 and brown = group 2). The left panel shows the mean relative proportions (%) of each KO, while the right panel presents differences in mean proportions with 95% confidence intervals.

Ten KEGG orthologs (KOs) between the studied bacterial groups were highly predicted and enriched according to potentials analysis inferred from taxonomic composition (Figure 8). Of these, K08640 (zinc D-Ala-D-Ala carboxypeptidase), K00657 (diamine N-acetyltransferase), K06147 (ATP-binding cassette, subfamily B) and K07024 (sucrose-6-phosphatase) had the strongest significant and potential enrichment patterns between groups with a mean proportion of approximately 0.30%, indicating considerably greater relative abundances in group 1. Overall, the findings show clear predicted functional changes in the mix of KEGG orthologs between the two groups, with K06147 and K07024 making the most contributions to the observed divergence.

## 3. Discussion

Acacia species have a wide range of ecological adaptation and a high level of resistance to salinity and drought. These multipurpose trees yield vital items such as fuelwood, timber, gums, tannins, and feed. In dry areas, increasing the use of underused species in restoration projects could promote sustainable development and biodiversity conservation [38,39]. Two Acacia trees were selected for the current study (*Acacia gerrardii* Benth and *Acacia raddiana* Savi), for possible contribution in increasing vegetation cover in arid landscapes, thereby reducing soil erosion, enhancing carbon sequestration, and supporting ecosystem restoration and sustainability. Soil microbiome facilitate the plant nutrient acquisition, supporting their growth as a plant growth promoting rhizobacteria (PGPR) [40,41,42]. Plant growth-promoting rhizobacteria, mycorrhizal fungi, endophytes, actinomycetes, protozoa, and nematodes interact dynamically with plant roots and are essential to plant growth and health. Both plant species and soil properties have a significant impact on these plant–microbe interactions, which can be symbiotic, commensal, or competing. In addition to improving nutrient uptake, beneficial soil microbes also promote plant development, increase stress tolerance, inhibit diseases, and aid in carbon sequestration and phytoremediation [43,44,45]. Differences in microbial community composition among soil compartments may reflect variation in local environmental conditions, including nutrient availability, pH, organic matter, salinity, soil texture which is recognized as an important driver of microbiome assembly. Similar patterns have been reported in previous studies linking soil nutrient status with shifts in microbial diversity and functional groups. Nevertheless, the present study did not directly test associations between soil physicochemical variables and microbial community structure. Consequently, any nutrient-related interpretation should be regarded as hypothetical and supported primarily by evidence from the literature rather than by direct statistical evidence from this dataset [46,47].

In the present study, plant species-level comparisons were conducted to investigate host-associated differences in soil and plant parameters. However, because individual sampling locations may differ in local environmental conditions, the observed patterns should be interpreted as reflecting the combined influence of host species identity and site-specific environmental variation. These analyses provide a more appropriate test of the biological hypothesis than location-specific comparisons alone. Soil samples in group 2 displayed significant and high availability from P, Mn, Zn and Cu elements. The plant samples from *A. gerrardii* Benth (group 1) manifested high and significant content from Mn and Ca when compared with and *A. raddiana* Savi (group 2). The bacterial genus *JG30-KF-CM45* and the fungal genera (i.e., *Fusarium*, *Didymella* and *Pithoascus*) in addition to phyla such as Gemmatimonadota and Actinobacteriota were the most dominant microorganisms from the Acacia-associated rhizosphere communities in these soil samples. Although our previous study [48] characterized rhizosphere microbiomes associated with desert shrubs (*Zygophyllum simplex* and *Haloxylon salicornicum*) using a comparable sequencing framework, the present work extends this knowledge by focusing on native Acacia tree species within a protected arid reserve and by integrating microbial community composition with both soil physicochemical properties and leaf nutrient profiles. These additions provide novel insights into the ecological drivers of plant–soil-microbiome interactions in desert woody ecosystems. In a design experiment, Li et al., (2025) [49], evaluated the effect of exogenous addition of different carbon sources and different salinization degrees on the structure and microbial community of soils in cotton fields. At the genus level, *Arthrobacter* (11.61%), *JG30-KF-CM45* (5.95%), *Vicinamibacterales* (3.38%), *Vicinamibacteraceae* (2.86%), and *Sphingomonas* (1.98%) accounted for 25.33% of the total bacterial communities. Likewise, *Lectera* (15.68%), *Cladosporium* (12.18%), *Alternaria* (10.81%), *Chactomium* (9.36%), and *Fusarium* (8.15%) were the top five dominating genera by relative abundance at the genus level, making up 56.24% of the total fungal communities. Dai et al. [50], investigated the relationship among the diversity, function and abundance of soil microbiomes and the metallic micronutrients (Fe, Mn, Cu, Zn, Mo and Ni) across 180 sites in China. The available Fe (0.27), Cu (0.15), Mn (0.16), Ni (0.15), and Zn (0.28), all showed positive correlations with bacterial total abundance, whereas the fungal total abundance was only favorably linked with available Zn (0.18), Mo (0.17), and total Zn (0.17). Moreover, bacterial total abundance was positively linked with soil macronutrients and pH (except from soil C:N), but fungal richness was only affected by total P and soil pH.

According to the findings reported by Shen et al. [51], four of the top 20 genera involving *Elsterales*, *Streptomyces*, unidentified *JG30-KF-CM45*, and unidentified *JG30-KF-AS9*, were considerably enriched in the fertilizer-reducing soil treatments, whereas the relative abundance of *Arthrobacter* decreased in the fertilizer-reducing treatments. In a temperate steppe, a factorial nutrient-addition experiment revealed that nitrogen enrichment suppressed oligotrophic taxa (Acidobacteria, Chloroflexi, and Verrucomicrobiota) while favoring copiotrophic bacterial groupings (Proteobacteria, Firmicutes, and Bacteroidota). The only therapy that considerably boosted bacterial alpha diversity was phosphorus supplementation, which also decreased the proportion of phoD-containing bacteria by easing phosphorus limitation. The makeup of the bacterial community was more affected by variations in soil nutrient availability (NO_3_^−^-N, NH_4_^+^-N, accessible P, and dissolved organic carbon) than by changes in pH brought on by nitrogen addition. In general, soil bacterial populations and functions were altered by nutrient enrichment, particularly phosphorus availability, which had significant effects on soil biogeochemical cycle [52].

While plant-associated microbial communities improve plant growth, stress tolerance, and ecosystem resilience, climate change presents significant hurdles to agricultural sustainability. Advanced genomic and amplicon-sequencing approaches are improving our understanding of soil microbial diversity, plant–microbe interactions, and their critical roles in nutrient cycling, carbon sequestration, and adaptation to changing environmental conditions [53,54]. Currently, the bacterial genera such as *Rubrobacter*, *Kribbella*, *Saccharopolyspora* and the fungal genera like *Metarhizium*, *Cordyceps*, *Scutellinia*, *Arcuadendron* and *Clonostachys* were the most prevalent microflora in all soil samples. In tomato plants, potential plant-beneficial bacterial (PBB) communities revealed compartment-specific distribution patterns, with the phyllosphere displaying the lowest richness and the rhizosphere the highest diversity and abundance. The rhizosphere-associated PBBs were dominated by Actinobacteria and Rubrobacteria, while the phyllosphere was dominated by Actinobacteria and Bacilli. *Rubrobacter*, *Blastococcus*, and *Nocardioides* were the most prevalent taxa in all compartments at the genus level [55]. Otherwise, the most prevalent fungal phyla in the strawberry rhizosphere soil were Monoblepharomycota, Glomeromycota, and Mucoromycota, while the most common bacterial taxa were Proteobacteria, Bacteroidota, and Acidobacteriota [56]. Under continual wheat, the community’s composition also changed significantly, with Actinobacteria and Bacteroidetes having higher relative abundances while Acidobacteria were less common. Crop rotation and nutrient management have a significant impact on soil microbial diversity, community structure, and related biogeochemical processes, as demonstrated by the additional promotion of Bacteroidetes by fertilization. Ninety percent of the soil samples contained the core bacterial taxa *Bradyrhizobium*, *Rubrobacter*, *Friedmanniela*, and *Marmoricola* [57].

In the current study, Actinobacteriota, Chloroflexi, and Proteobacteria were the dominant phyla in the bacterial communities whereas Ascomycota and Basidiomycota presented the maximum enrich fungal phyla. In soils derived from *Tamarix aphylla* and *Halopeplis perfoliata*, amplicon-sequencing study of 16S and ITS rRNA genes showed that Actinobacteriota and Proteobacteria were the predominant bacterial phyla, while Ascomycota and Basidiomycota were the predominant fungus communities [58]. Additionally, the bacterial communities’ richness and evenness based on α- and β-diversity were higher in group 2 (*A. raddiana* Savi) than group 1 (*A. gerrardii* Benth), while fungal communities were more variable in group 1 (*A. gerrardii*) vs. group 2 (*A. raddiana*), without significant difference. Bacterial α-diversity was connected to soil crusts and β-diversity to rhizosphere habitats, indicating a considerable difference in microbial community structure between the rhizosphere and soil crust compartments. Analyses of fungal diversity also revealed clear compartment-driven community clustering, emphasizing robust habitat specific microbial assembly in soils associated with halophytic plants [58]. While management intensity greatly influenced both fungal and prokaryotic communities, local environmental factors (soil characteristics and topography) caused greater variation in soil microbial diversity across 86 permanent grasslands in Switzerland than management approaches. Coprophilous and nitrogen-cycling taxa were more prevalent in intensively managed and fertilized grasslands, while organic farming had very little direct impact on the structure of fungal communities [59].

The most putative abundant enzymes with higher alteration in bacterial communities under Acacia plants are EC:3.4.17.14 (Zinc D-Ala-D-Ala carboxypeptidase), EC:3.5.1.4 (Amidase), EC:2.5.1.7 (UDP-N-acetylglucosamine 1-carboxyvinyltransferase), and EC:3.2.1.1 (Alpha-amylase). However, these predictions reflect inferred functional potential derived from community composition and do not constitute direct evidence of gene expression, enzyme abundance, or enzymatic activity. Therefore, these findings should be interpreted as indicative of potential ecological functions that require validation through metagenomic, metatranscriptomic, or biochemical analyses. Soil microbial amidase drives the nitrogen cycle and detoxifies contaminants by breaking down non-peptide amide bonds into carboxylic acids and ammonia. Alpha-amylase in soil functions as an extracellular enzyme that breaks down complex starches into simpler sugars, driving the carbon cycle and providing energy for soil microbes. Key soil microbes like Bacillus species release this enzyme to digest plant debris [60,61].

The putative activity of soil enzymes involved in the cycling of carbon, nitrogen, and phosphorus (β-glucosidase, N-acetyl-β-glucosaminidase, and acid phosphomonoesterase) closely matched patterns of soil nitrogen supply across fertilizer treatments and crop rotations. Compared to diverse rotations, continuous wheat farming decreased soil bacterial α-diversity, which further decreased after fertilizer was applied [57]. To maintain cell survival in a variety of settings, bacteria employ selective membrane trafficking techniques. The largest and most varied superfamily of membrane transport systems are ATP-binding cassette (ABC) transporters (K06147 pathway, ATP-binding cassette), which carry substrate across the cytoplasmic membrane using the energy from ATP hydrolysis. Numerous cell processes, such as cell division and morphogenesis, gene control, surface motility, chemotaxis, and interspecies competition, depend on these transporters, which also import nutrients and export chemicals [62,63]. In the present study, the absence of a bulk-soil reference which precludes direct assessment of rhizosphere-specific microbial recruitment relative to the surrounding desert soil, is one of the study’s limitations. The current sampling design does not permit causal inference regarding the effect of Acacia species identity on rhizosphere microbial assemblages. Therefore, observed microbiome differences may reflect not only host-associated factors but also spatial and environmental heterogeneity. Consequently, the findings should be interpreted as an exploratory comparison of two naturally occurring Acacia populations rather than definitive evidence of a host-species effect. Future studies employing co-located sampling designs and detailed environmental measurements are required to disentangle species-specific and site-related influences on rhizosphere microbial communities. A future study should take in consideration a bulk (non-rhizosphere) soil comparator for general soil background and detailed environmental measurements to disentangle species-specific and site-related influences on rhizosphere microbial communities.

## 4. Materials and Methods

### 4.1. Area of Study and Samples Collection

**Figure 9:**
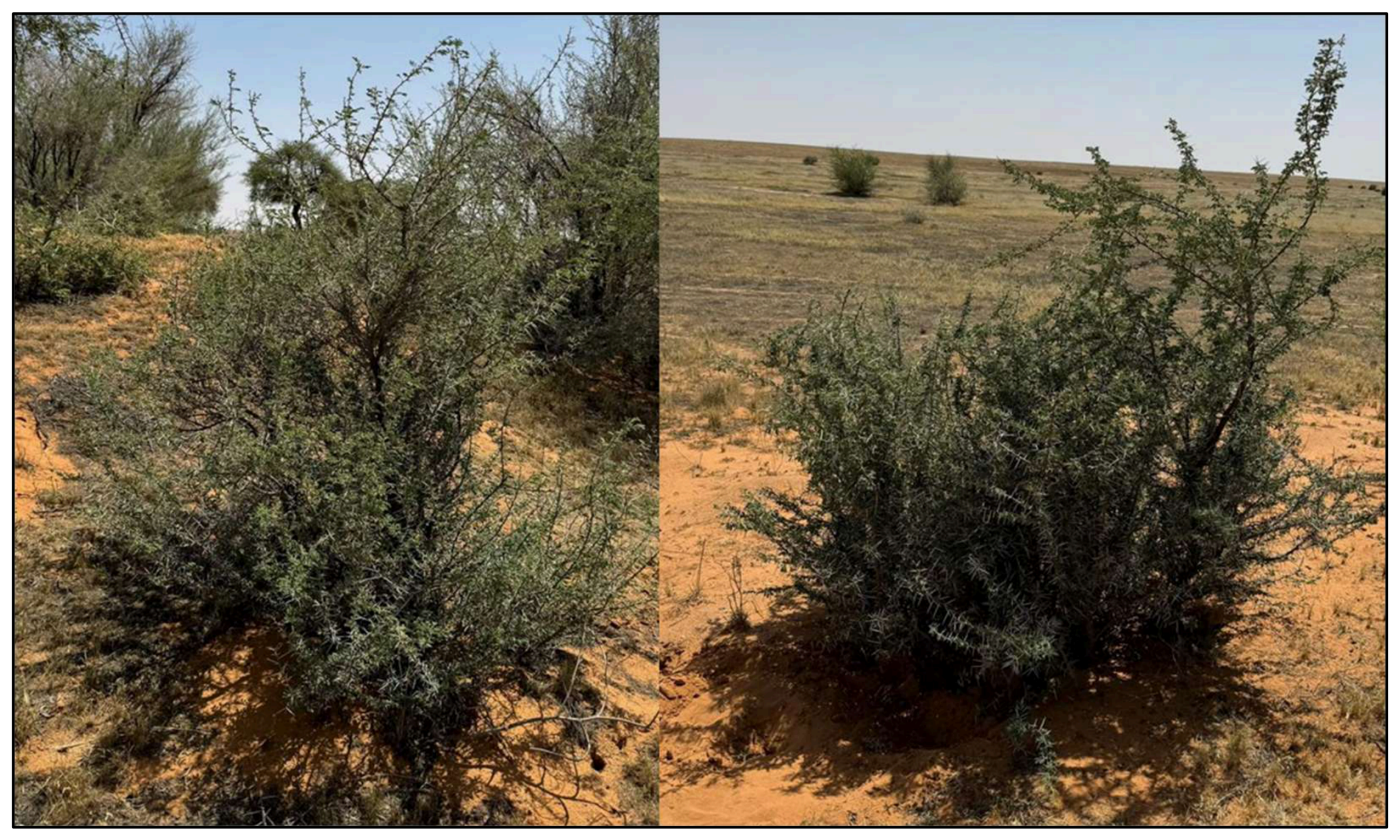
Acacia trees in the Imam Turki bin Abdullah (ITBA) Royal Natural Reserve.

By royal order, the Imam Turki bin Abdullah (ITBA) Royal Natural Reserve Development Authority was issued in 2018 to safeguard the Kingdom’s biodiversity and natural environment through an integrated system that forbids logging, poaching, and overgrazing while promoting sustainable ecotourism. The reserve’s distinctive plant and animal richness, as well as its rich cultural and historical legacy, make it one of the most well-known natural places in the Kingdom. It is situated in the northeastern part of the country, inside the administrative borders of many regions, including Eastern Province, Jawf, Qassim, Hail, and Northern Borders. The ITBA Royal Natural Reserve is located between latitudes 26°45′ and 30°15′ N and longitudes 40° and 45° E with valley length reached 40 km long whereas our samples are taken [5]. Fifteen leaf samples from *Acacia gerrardii* Benth and *Acacia raddiana* Savi [64,65] (2–3 year-old shoots) and their corresponding rhizosphere soil samples were collected from down towards up of the valley in the reserve and transported to the Soil and Biotechnology Laboratories at Qassim University for subsequent analyses. The unequal sample size reflects the natural distribution, age, size and availability of mature Acacia individuals across the study transects within the sampling area. The locations of plant and soil samples are presented in Table 3. In soil samples, three samples from the rhizosphere area behind each plant were gathered and mixed together to form a homogenized composite samples from each location. In addition, plant samples were combined from leaves and branches of each plant to prepare one composite sample from plant (Figure 9). Each composite sample was subsequently divided into three equal aliquots and processed independently as technical replicates.

### 4.2. Soil Physicochemical Properties

The titration method was utilized to quantify soluble cations and anions using saturated soil paste extracts; calcium and magnesium were extracted using an EDTA solution; carbonate were analyzed using hydrochloric acid. Elements such as Fe, Mn, Zn, Ca, Cu, S, CaCo_3_ and Soil texture were measured using ICP Spectrometer-iCAP7000 Series (Thermo Fisher Scientific, Cambridge, UK), based on AOAC Official method of analysis [66] (Table 4). Additionally, phosphorus (P), and magnesium (Mg) were measured using spectrophotometer (APEL, PD-303UV, Kawaguchi, Japan) in accordance with AOAC Official method [66]. The soluble sodium (Na) and available Potassium (K) were assessed by Flame photometer (BWB, Newbury, UK) in compatible with AOAC Official method. The pH in soil suspension was measured at a ratio of 1:2.5 using a type of pH meter (HACH-HQ Series Multi Meter, Loveland, CO, USA), total N was determined using the Kjeldahl method (VELP- UDK 149 Automatic Distillation unit, Lombardy, Italy), EC (dSm^−1^), and Total Dissolved Solids (TDS) were measured in the saturated soil paste extract using a type of EC meter (HI991300-pH/EC/TDS Meter, Limena, Italy).

### 4.3. The Soil and Plant Contents from Nutritional Elements

Soil and plant samples were evaluated for their chemical contents of the nutritional elements. The levels of nutrient elements (N, P, K, Fe, Mn, Zn and Cu) in soil and plant samples, in addition to Mg and Ca in plant samples, were assessed according to Page 1982 [67].

### 4.4. DNA Extraction, PCR Amplification and DNA Library Construction

The DNeasy Power Max soil kit (cat. no. 12988-10, Qiagen, Hilden, Germany) was utilized in accordance with the manufacturer’s instructions to extract the total genomic DNA from the microbial communities existed in the soil samples. The NanoDrop device (NanoDrop ND-1000 Spectrophotometer, Thermo Scientific, Wilmington, DE, USA) was applied to evaluate the concentration and purity of separated DNA. In the first PCR round, the PCR-amplified using the primers 341F 5′ CCTACGGGNGGCWGCAG 3′ and 805R 5′ GACTACHVGGGTATCTAATCC 3′ for the bacterial 16S rRNA (V3-V4 amplicons) to amplify ~ 460 bp fragment, while in the second round, i5 index PCR primer: 5′AATGATACGGCGACCACCGAGATCTACACXXXXXXXXACACTCTTTCCCTACACGACGCTCTTCCGATCT3′ and i7 index PCR Primer: 5′CAAGCAGAAGACGGCATACGAGATXXXXXXXXGTGACTGGAGTTCAGACGTGTGCTCTTCCGATCT3′ were applied. Furthermore, ITS2F 5′ GTGAATCATCGAATCTTTGAA 3′ and ITS2R 5′ TCCTCCGCTTATTGATATGC 3′ were used to amplify the fungal ITS2 region. A volume of 50 μL was used for PCR amplification, using 50 ng gDNA (1 μL), 25 μL 2 × PCR master mix (DreamTaq PCR Master Mixes, ThermoFisher Scientific, Waltham, MA, USA), 0.5 μL each of forward and reverse primers (0.2 μM), and 23 μL of dH_2_O. All PCR amplifications were performed in the thermal cycler (Bioer, China) after 2 min of denaturation at 95 °C, 35 cycles of 60 s at 95 °C, 30 s of annealing at 50 °C, 90 s at 72 °C, and a final extension of 7 min at 72 °C.

### 4.5. Amplicon Sequencing and Analysis

The amplified PCR products were subjected to purification and library building. The samples libraries were built using Zymo Quick-16S Plus NGS Library Prep Kit (V4). Furthermore, amplicons were sequenced on an Illumina MiSeq platform to generate 2 × 300 bp paired-end reads. Following that, raw sequencing data from both the 16S rRNA gene and ITS libraries were demultiplexed and processed in QIIME2 (v2024.10) [68] using the DADA2 plugin [69], to generate Amplicon Sequence Variants (ASVs) as the final output. Reads were quality filtered and trimmed, denoised through error correction, merged to reconstruct full amplicon sequences, and screened for chimeras using the consensus method implemented in DADA2. The resulting ASVs were used for subsequent taxonomic and microbial community analyses.

Assigning taxonomy to the sequences to explore the taxonomic composition was performed using SILVA 138 NR99 [70] reference database for bacterial 16S rRNA gene sequences while fungal ITS sequences were classified against the UNITE database (version 8.0) [71]. Taxonomic assignments were generated using pre-trained Naive Bayes classifier with a confidence threshold of 0.70. Following quality filtering and denoising with DADA2, an ASV feature table comprising 15 samples, 42,632 ASVs, and 293,669 total sequences was generated. The post-denoising sequencing depth ranged from 15,000 to 34,527 reads per sample (median = 18,234 reads). Samples were rarefied to 4000 reads per sample prior to alpha-diversity analysis. For species abundance heatmap, R language tools (version 4.1.2) were applied to perform the heatmap analyses [72], whereas alpha and beta diversity were calculated through the multi-omics bioinformatics platform QIIME 2 through Chao1, Shannon, Simpson, Ace, rarefaction curve, boxplot analysis, principal Coordinate Analysis (PCoA) [68,73,74,75,76]. For bacterial communities, representative ASV sequences were aligned using MAFFT [77] and a rooted phylogenetic tree was generated using FastTree [78] through the QIIME2 workflow. Weighted UniFrac distances [79] were calculated from the rooted phylogeny and rarefied ASV table and visualized using principal coordinates analysis (PCoA). For fungal ITS communities, Bray–Curtis dissimilarity was used to assess beta diversity because ITS sequences do not reliably support the generation of a phylogenetically informative tree suitable for UniFrac-based analyses. PERMANOVA [80] was performed to test the differences in microbial community composition among groups, followed by PERMDISP analysis [81] to assess whether significant PERMANOVA results were confounded by heterogeneity in group dispersion.

The predictive functional profiling (KEGG Orthologs (KOs), Enzyme Commission (EC)) of microbial communities (Bacteria 16S rRNA) was inferred from the representative ASV sequences using PICRUSt2 (version v2.6.3) [82]. Differences in predicted microbial functional categories among sampling sites were assessed using the Statistical Analysis of Metagenomic Profiles (STAMP) software (v2.1.3) [83], whereas differential abundance analysis in heatmap was performed by non-parametric Kruskal–Wallis test with Benjamini–Hochberg FDR correction (q < 0.05). In addition, effect sizes were computed using the difference in proportions supplied by STAMP in order to assess the extent of variations between groups. The generated datasets from the amplicon sequences were deposited at the NCBI repository under the SRA data accession number PRJNA1524290.

### 4.6. Data Statistical Analysis

One-way analysis of variance (ANOVA) and Tukey’s honestly significant difference (HSD) post hoc test were used for multiple comparisons. Student’s *t*-test was used to measure differences in the group-level comparisons. For each plant, three soil/plant subsamples were collected and pooled to generate a composite sample, which was subsequently partitioned into three aliquots and analyzed as technical replicates. Differences were considered statistically significant at *p* < 0.05 [84].

## 5. Conclusions

In northern Saudi Arabia, the Imam Turki bin Abdullah Royal Reserve is a significant protected ecosystem with extensive Acacia-dominated habitats that sustain ecosystem function and biodiversity. Soil microbiota diversity may enhance nutrient acquisition, improving soil fertility, and increasing plant tolerance to environmental stress. Soil and plant samples (between the two groups) varied in their contents from nutrients, reflecting possible role of the soil microorganisms in nutrient elements availability. Actinobacteriota, Chloroflexi, Proteobacteria and Gemmatimonadota, in addition to Ascomycota and Basidiomycota recorded the maximum enrich phyla under Acacia plants in the reserve. Bacterial genera such as *Rubrobacter*, *Kribbella*, *Saccharopolyspora* and *JG30-KF-CM45* were the most abundant bacteria, whereas *Metarhizium*, *Cordyceps*, *Acremonium*, *Lepidosphaeria*, *Blastomyces*, *Trichothecium* and *Gibellulopsis* displayed the most prevalent fungal microflora. Furthermore, enzymes like Zinc D-Ala-D-Ala carboxypeptidase, Diamine N-acetyltransferas, Serine-type D-Ala-D-Ala carboxypeptidase, Amidase, UDP-N-acetylglucosamine 1-carboxyvinyltransferase, and Alpha-amylase showed a significant alteration and a relative abundances among the two studied Acacia species. A limitation of the present study is the absence of a bulk-soil reference, which precludes direct assessment of rhizosphere-specific microbial recruitment relative to the surrounding desert soil. Furthermore, environmental heterogeneity and site-specific factors may have contributed substantially to the detected patterns. In arid environments, these microbial interactions are especially crucial for Acacia productivity, survival, and ecosystem restoration.

## Figures and Tables

**Table 1:** The available soil nutrients under *Acacia gerrardii* Benth (group 1) and *Acacia raddiana* Savi (group 2) plants in the Imam Turki bin Abdullah (ITBA) Royal Natural Reserve.

Group	Soil Samples	N (%)	P (ppm)	K (ppm)	Fe (ppm)	Mn (ppm)	Zn (ppm)	Cu (ppm)
Group 1	A1	0.146	9.55	68.00	2558.67	22.30	13.66	2.20
	A2	0.128	1.57	40.33	3865.67	34.29	18.22	4.28
	A3	0.137	14.26	42.33	3723.33	68.28	28.82	5.56
	A4	0.171	4.48	33.67	2775.33	25.10	16.56	2.05
	A5	0.180	17.01	52.33	1800.33	23.73	10.73	3.38
	A6	0.152	8.16	59.67	2560.00	22.25	15.92	4.54
	A7	0.121	5.89	35.33	2933.00	25.72	15.47	5.04
	A8	0.157	27.57	79.67	3686.00	36.36	19.46	4.05
	A9	0.149	11.41	53.67	2796.33	27.33	15.03	4.85
Group 2	A10	0.197	17.03	39.33	3714.67	41.23	18.34	5.44
	A11	0.107	17.79	40.33	3795.00	53.41	24.54	6.59
	A12	0.117	35.17	105.67	3167.67	69.64	30.81	7.20
	A13	0.122	24.34	53.67	3269.33	52.20	27.92	6.00
	A14	0.134	11.93	71.67	2803.33	62.81	29.04	5.29
	A15	0.116	25.31	120.33	3078.67	59.17	21.09	6.133
Group 1	A (1–9)	0.15 ± 0.02 a	11.1 ± 7.82 b	51.67 ± 15.51 a	2966.52 ± 676.12 a	31.71 ± 14.6 b	17.1 ± 5.07 b	3.99 ± 1.23 b
Group 2	A (10–15)	0.13 ± 0.03 a	21.93 ± 8.17 a	71.83 ± 34.28 a	3304.78 ± 382.35 a	56.41 ± 9.81 a	25.29 ± 4.86 a	6.11 ± 0.71 a

Different letters in the same column mean significant differences (*p* ≤ 0.05).

**Table 2:** The available plant nutrients in *Acacia gerrardii* Benth (group 1) and *Acacia raddiana* Savi (group 2) leaves in the Imam Turki bin Abdullah (ITBA) Royal Natural Reserve.

Group	Plant Samples	N (%)	P (ppm)	K (ppm)	Fe (ppm)	Mn (ppm)	Zn (ppm)	Cu (ppm)	Mg (ppm)	Ca (ppm)
Group 1	A1	3.67	1714.33	9020.33	254.08	35.50	22.07	5.43	2115.00	5426.00
	A2	1.21	905.21	6467.00	146.73	23.60	19.93	6.63	1867.00	5308.67
	A3	2.21	758.12	6813.67	169.37	37.25	15.47	8.80	2125.67	6616.33
	A4	2.64	911.51	6597.00	235.54	54.78	14.00	5.74	2224.33	6196.67
	A5	2.62	1072.67	6733.00	142.06	31.83	13.19	6.21	1926.00	5829.00
	A6	2.66	1608.72	5423.00	168.97	27.93	17.81	7.09	1021.00	6378.00
	A7	2.61	707.40	6497.33	126.51	23.94	14.10	7.29	1162.33	5813.33
	A8	2.07	722.00	7823.67	159.44	27.03	11.27	8.58	2170.67	6417.33
	A9	2.53	715.27	6890.33	132.28	28.86	13.70	10.86	1968.33	6228.67
Group 2	A10	2.47	898.52	6704.33	134.93	27.14	11.19	8.05	2165.33	5329.67
	A11	2.72	837.77	5544.33	136.47	27.04	8.33	6.53	2313.33	5121.00
	A12	2.61	1243.57	8644.33	126.53	13.45	5.40	6.59	1055.67	5221.00
	A13	2.11	904.04	6947.67	131.27	22.43	16.34	6.18	2165.33	5325.33
	A14	2.16	502.82	6418.33	122.44	20.97	14.75	8.55	2339.67	5620.67
	A15	2.03	475.130	6365.67	154.70	22.88	17.40	7.54	1913.33	5424.67
Group 1	A (1–9)	2.47 ± 0.65 a	1010 ± 390 a	6920 ± 100 a	170.55 ± 44.88 a	32.3 ± 9.65 a	15.73 ± 3.5 a	7.4 ± 1.73 a	1840 ± 440 a	6020 ± 450 a
Group 2	A (10–15)	2.35 ± 0.29 a	810 ± 290 a	6770 ± 1030 a	134.39 ± 11.24 a	22.32 ± 5.03 b	12.24 ± 4.75 a	7.24 ± 0.95 a	1990 ± 480 a	5340 ± 170 b

Different letters in the same column mean significant differences (*p* ≤ 0.05).

**Table 3:** Locations of collected soil and plant samples from Acacia plants in the Imam Turki bin Abdullah Royal Natural Reserve.

N		Sample Code	Location (Longitude and Latitude)
1	Group 1 (*Acacia gerrardii Benth*)	A1	28°15′07.8″ N 43°33′09.2″ E
2		A2	28°15′07.3″ N 43°33′13.5″ E
3		A3	28°15′07.9″ N 43°33′16.6″ E
4		A4	28°13′44.8″ N 43°30′54.4″ E
5		A5	28°13′45.7″ N 43°30′53.5″ E
6		A6	28°13′42.9″ N 43°30′49.9″ E
7		A7	28°14′17.2″ N 43°35′27.1″ E
8		A8	28°14′14.3″ N 43°35′32.4″ E
9		A9	28°14′14.4″ N 43°35′34.9″ E
10	Group 2 (*Acacia raddiana Savi*)	A10	28°12′13.1″ N 43°38′10.9″ E
11		A11	28°12′05.3″ N 43°38′23.6″ E
12		A12	28°11′56.7″ N 43°38′35.8″ E
13		A13	28°10′11.4″ N 43°43′23.0″ E
14		A14	28°10′10.6″ N 43°43′23.7″ E
15		A15	28°10′11.2″ N 43°43′24.7″ E

**Table 4:** Soil chemical properties of samples gathered from the rhizosphere of Acacia trees.

N	Properties	Value
Physical properties		
1	Sand (%)	97.30%
2	Silt (%)	1.42%
3	Clay (%)	1.28%
4	Soil Texture	Sand
5	Organic matter (O.M, %)	0.29%
Chemical properties		
6	Magnesium (Mg)	947 ppm
7	Calcium (Ca)	2040 ppm
8	Sulphur (S)	49.63 ppm
9	Boron (B)	0.6 ppm
10	Calcium Carbonate (%)	0.92%
11	pH	8.14
12	Electrical Conductivity (EC)	0.39 mS/cm
13	Total Dissolved Solids (TDS)	197.4 ppm
14	Soluble sodium (Na)	13.2 ppm

## Data Availability

Data available within the manuscript or in the Supplementary Materials. The generated datasets from the amplicon sequences are available in the NCBI repository, under the SRA data accession number PRJNA1524290.

## Supplementary Materials

The following supporting information can be downloaded at: https://www.mdpi.com/article/10.3390/plants15193023/s1, File S1: Bacterial genus_percentages.xls: t; File S2: Fungal genus_percentages.xls; Table S1: Data statistics of the quality control (Bacteria); Table S2: Data statistics of the quality control (Fungi).
