# Supplementary material for: Characterization of Soil Microbial Communities Associated with the Sustainability of Acacia Species in the Imam Turki Bin Abdullah Royal Reserve, Saudi Arabia
Source: Plants (Basel). 2026 Oct 2;15(19):3023. doi: 10.3390/plants15193023 (PMC13667072; doi:10.3390/plants15193023)
Supplement: Supplementary file 1 [file plants-15-03023-s001.zip › Supplementary materials.pdf]

Table S1. Data statistics of the quality control (Bacteria)

Sample_ID	PE_Reads	Clean_Tags	AvgLen(bp)	GC_Content	≥Q20	≥Q30
A1	251918	250278	249	0.59	97.42	92.67
A2	212034	210408	249	0.61	97.29	92.47
A3	187744	186504	249	0.60	97.41	92.67
A4	255640	253966	249	0.60	97.47	92.82
A5	446730	443996	249	0.63	97.71	93.31
A6	220226	219060	249	0.60	97.58	93.00
A7	204336	202986	249	0.60	97.68	93.25
A8	216638	215164	249	0.59	97.37	92.63
A9	210896	209552	249	0.59	97.41	92.65
A10	231930	230294	249	0.60	97.36	92.61
A11	237298	235624	249	0.60	97.35	92.57
A12	261894	260254	249	0.60	97.45	92.76
A13	296932	295142	249	0.61	97.57	92.94
A14	203132	201972	249	0.60	97.56	92.95
A15	230576	229216	249	0.60	97.66	93.20

Sample_ID	PE_Reads	Clean_Tags	AvgLen(bp)	GC_Content	≥Q20	≥Q30
A1	146346	145662	249	0.58	98.43	95.03
A2	114628	114002	249	0.54	98.36	94.92
A3	181058	180102	249	0.62	98.07	94.41
A4	184252	183332	249	0.64	97.84	94.13
A5	250476	249288	249	0.65	98.53	95.35
A6	145018	144394	249	0.60	98.03	94.26
A7	160882	160122	249	0.63	98.13	94.49
A8	164984	164178	249	0.61	98.25	94.64
A9	167464	166676	249	0.61	98.43	95.11
A10	184170	183304	249	0.61	98.40	95.08
A11	171946	171064	249	0.59	98.44	95.04
A12	143434	142698	249	0.57	98.40	95.02
A13	174168	173440	249	0.64	98.50	95.18
A14	147120	146350	249	0.59	98.29	94.85
A15	149680	149064	249	0.64	98.13	94.61

Table S2. Data statistics of the quality control (Fungi)

PE Reads: the PE reads obtained from the sequencing platform. Clean Tags: tags following QC. AvgLen: the average length of the Clean Tags. GC (%): the percentage of G and C. Q20%&Q30%: the percentage of bases with a quality score equal to or higher than 20 (error rate <1%) and 30 (error rate <0.1%). Clean(%): the number of Clean Tags take up of the number of Raw PE.
